# A Pose Initialization Method for Unmanned Vehicles Based on an Improved Siamese Neural Network and Multi-Stage Probabilistic Registration Localization

**DOI:** 10.3390/s26113335

**Published:** 2026-05-24

**Authors:** Jian Yang, Biao Chen, Weiye Shen, Xiaobin Xu

**Affiliations:** 1 College of Mechanical Engineering, Yangzhou University, Yangzhou 225127, China; MZ120230911@stu.yzu.edu.cn (B.C.); MZ120250863@stu.yzu.edu.cn (W.S.); 2 College of Mechanical and Electrical Engineering, Hohai University, Changzhou 213200, China; xxbtc@hhu.edu.cn

**Keywords:** adaptive Monte Carlo localization, localization, normal distributions transform, Siamese neural network

## Abstract

In satellite-denied environments, conventional localization methods struggle with rapid pose initialization due to the absence of global positioning data. To address this challenge, this study presents a high-precision pose initialization framework based on a Siamese Neural Network (SNN) and multi-stage probabilistic registration localization. First, the SNN improved by Convolutional Block Attention Module (CBAM) matches features between real-time radar point clouds and prior map slices, producing candidate positions based on similarity scores. Then, Adaptive Monte Carlo Localization (AMCL) performs probabilistic matching among these candidates to identify the correct slice and refine the position accuracy from tens of meters to meter-level, along with an approximate orientation estimate. Finally, the Normal Distributions Transform (NDT) is applied for point cloud registration, achieving centimeter-level pose estimation. The proposed method is evaluated on self-collected medium-scale and large-scale maps. Experimental results show that the SNN effectively identifies the correct map slice, which is further refined by AMCL and NDT to achieve centimeter-level position accuracy and sub-degree orientation accuracy. The multi-stage method achieves localization success rates of 99% on both 200 × 100 m and 300 × 200 m regions, with distance RMSEs of 0.175 m and 0.348 m, and orientation RMSEs of 0.149° and 0.437°, respectively. Evaluations on the KITTI dataset further demonstrate robust initialization performance in complex outdoor environments. The proposed framework provides a reference for high-precision pose initialization in large-scale satellite-denied scenarios.

## 1. Introduction

Unmanned vehicles, owing to their intelligence, efficiency, and cost-effectiveness, have been widely adopted in fields such as intelligent logistics, smart transportation, precision agriculture, and defense applications. However, when operating in complex environments like indoor spaces, densely populated urban districts, or vegetation-covered areas, their localization is challenged by signal degradation or even the unavailability of the Global Navigation Satellite System (GNSS). In such GNSS-denied environments, pose initialization serves as a critical prerequisite step for unmanned vehicles, which directly impacts the accuracy and robustness of subsequent positioning and navigation processes [1,2]. Incorrect initialization adversely affects iterative positioning performance, increasing the risk of convergence failure or higher computational cost. These errors further propagate to planning and control modules, degrading overall system performance. To address localization in GNSS-denied environments, probabilistic filtering-based methods, feature-based matching methods, and deep learning-based methods have been widely explored for pose initialization [3,4,5], differing in how sensor data are processed. From the perspective of sensing modality, pose initialization methods are primarily categorized into vision-based and LiDAR-based approaches. Vision-based methods use cameras to capture artificial markers or to acquire environmental images, where feature-matching or deep learning algorithms are applied to identify keypoints in the scene. LiDAR-based methods depend on laser scanning of the surroundings, achieving initialization through point cloud matching or pre-constructed maps.

Vision-based methods can be marker-based, using artificial markers such as Quick Response (QR) codes, or markerless, relying on natural features in the environment. Marker-based methods are accurate and computationally efficient in positioning. For instance, a QR-based method [6] was introduced where the external QR code information and the internal encoder values are combined to refine pose estimation through extended Kalman filter. For large-scale or frequently updated environments, a smart artificial marker [7] was developed with sensing and ranging capabilities, enabling active self-reporting and more accurate visual localization. To enhance the robustness of indoor localization, Yu [8] presented a multi-source fusion framework integrating QR codes, Wi-Fi, Bluetooth Low Energy, and sensors. However, the application of marker-based methods is constrained by limited zones (only functional within marker-deployed areas), poor adaptability (requiring redeployment for scene modifications), and high costs of deployment and maintenance. Markerless methods, on the other hand, utilize natural environmental features such as wall corners, door frames, surface textures, or object edges. Keypoints and their descriptors can be extracted from images through feature detection algorithms. Similarity matching is then performed to determine the target’s position in the image. To achieve robust detection across multiple scales, Lowe [9] proposed Scale Invariant Feature Transform (SIFT), which constructs descriptors from local gradient information for image matching. To improve localization under illumination variation or occlusion, patch-NetVLAD [10] divides an image into patches and uses contextual cues. To enhance computational efficiency, a shared local feature strategy [11] was introduced, allowing feature reuse across images to eliminate redundant computation. Likewise, a prioritized matching approach [12] improves efficiency by exploiting visibility information from 3D reconstruction during 2D–3D feature matching. In recent years, deep learning-based visual localization methods have gained increasing attention, commonly employing convolutional neural networks (CNNs), Graph Neural Networks (GNNs), or transformers for image feature extraction and camera pose estimation. As for the CNNs, SuperPoint [13] adopts CNN to jointly compute pixel-level interest points and descriptors, with homographic adaptation improving cross-domain repeatability. Dusmanu [14] proposed D2-Net, where a single CNN simultaneously acts as dense feature descriptors and detectors, achieving robustness under day-night changes. To improve the accuracy, a partially differentiable CNN module [15] enables the generation of keypoints at a sub-pixel level through optimization with reprojection losses. To improve real-time localization performance in large environments, Sarlin [16] proposed a Hierarchical Feature Network (HF-Net) that integrates global retrieval and local matching within a coarse-to-fine pipeline. In terms of GNNs-based methods, SuperGlue [17], which matches local features by jointly estimating correspondences and rejecting non-matchable points; Angle-Annular-GNN [18], which efficiently learns robust geometric structural representations with annular feature extraction; and Sparse Spatial Scene Embedding-GNN [19], which encodes image embeddings into a pose graph for scene matching. In addition to CNNs and GNNs, recent works explore Transformer architectures for visual localization. For instance, Shavit [20] proposed using multi-headed Transformers to perform end-to-end absolute camera pose regression. Wang [21] integrated a hierarchical scene coordinate network with a Transformer to achieve robust scalability in large environments. EffLoc [22] leverages a hierarchical vision transformer with sequential group attention to enhance computational efficiency.

Laser-based localization methods estimate the pose by scanning environmental geometry and matching LiDAR data with an existing map. Laser-based methods are generally categorized into geometry-based and deep learning-based approaches. Typical geometric matching methods include Normal Distributions Transform (NDT) and Adaptive Monte Carlo Localization (AMCL). Derived from the classical Iterative Closest Point (ICP) algorithm, NDT improves robustness by replacing explicit point-to-point correspondences with probabilistic modeling of local geometric structures. NDT associates a piecewise normal distribution to the reference point cloud and finds the spatial transform that maximizes the probability of the source point cloud under this distribution. Several improvements have been made to enhance NDT performance. Multiscale Iterative NDT (MI-NDT) [23] improves efficiency in large-scale registration by dividing point clouds into multiple resolutions, allowing tolerance to larger initialization errors. Similarly, another variant [24] increases grid density at complex intersections and lowers it on open roads, balancing computational load and memory consumption in urban scenes. To reduce accumulated errors and drift probability, NDT-LOAM [25] introduces range weighting and surface features such as curvature to refine covariance estimation within NDT voxels. Its local geometric features are further used to refine poses after coarse NDT registration. To address large-scale topological localization, NDT-Transformer [26] compresses dense 3D point clouds into probabilistic NDT cells to describe geometric structures and employs a Transformer to learn global descriptors from these cells for location retrieval.

Adaptive Monte Carlo Localization (AMCL) applies a particle filter that relies on observed point clouds and their matching with a pre-built map. Particle weights are iteratively refined until convergence to the optimal pose estimation. Several improvements have been made to the AMCL. The Self-adaptive MCL (SAMCL) [27] introduces similar energy regions, where poses share comparable energy, to guide the distribution of global samples and achieve higher localization performance. To improve localization robustness in changing environments, a modified AMCL algorithm [28] incorporates object recognition and dynamic semantic map updating, while the Artificial Landmark Enhanced Localization AMCL (ALEL-AMCL) [29] integrates pre-positioned artificial landmark observations into the particle update process. To address the kidnapped robot situation with an unknown initial pose, one method [30] employs offline feature matching and particle swarm optimization, while another [31] integrates 2D laser and range finder information to achieve more reliable localization. Generally, NDT provides high accuracy and robustness but struggles with dynamic objects and feature-sparse environments, while AMCL is efficient and scalable but highly sensitive to pose initialization. On the other hand, deep learning-based LiDAR methods can automatically learn environmental features without manual parameter tuning, offering stronger robustness in dynamic environments. For instance, DOPNet [32] utilizes a graph convolutional network for feature extraction and a multilayer perceptron to predict spatial transformation. Furthermore, deformable kernels can be incorporated into graph convolutional networks [33] to enhance feature extraction in irregular and unstructured point clouds. PointLoc [34] leverages a PointNet-style network with self-attention to infer poses from a single LiDAR frame.

When prior pose information is unavailable, AMCL performs global localization by uniformly distributing particles across the entire map and iteratively updating their weights using motion and sensor measurements. However, its computational efficiency and localization reliability degrade as the map scale increases [30]. In large or structurally symmetric environments, the particle filter may suffer from slow convergence or ambiguity-induced degeneration, making global localization challenging [35]. Therefore, a reliable and efficient coarse localization mechanism capable of providing prior pose information is highly desirable for large-scale GNSS-denied environments. Sensor observations at the true location are expected to exhibit high similarity with the corresponding local map data. Siamese Neural Network, a deep learning architecture with twin branches sharing identical weights, can be employed to evaluate such similarities. It has been widely applied in comparison and matching tasks such as face verification [36], fingerprint-based authentication [37], and signature verification [38]. Inspired by this architecture, point cloud maps can be represented as images, and sensor-acquired point clouds can be compared with map slices through the Siamese network to estimate the robot’s position. Therefore, this paper introduces an innovative pose initialization algorithm that first employs a Siamese network for coarse localization and then integrates AMCL and NDT for fine pose refinement, enabling robust recovery from kidnapped situations.

The main contributions of this paper are as follows:

(1) A novel framework for coarse-to-fine localization is proposed, integrating a Siamese Neural Network for initial pose estimation and subsequent refinement with AMCL and NDT.
(2) By treating point cloud maps as images, coarse localization is achieved through the Siamese Neural Network that identifies the correct slice by computing similarity scores between sensor-acquired point clouds and the pre-existing map slices.
(3) The proposed approach achieves centimeter-level positional accuracy and sub-degree orientation accuracy under GNSS-denied conditions. It obtains localization success rates of 99% on both medium- and large-scale maps, with distance RMSEs of 0.175 m and 0.348 m, and orientation RMSEs of 0.149° and 0.437°, respectively.

The remainder of this paper is organized as follows: Section 2 introduces the overall framework of the algorithm, Section 3 presents experiments conducted on self-collected LiDAR point cloud maps and the KITTI dataset with result discussion, and Section 4 provides the concluding remarks.

## 2. Methods

**Figure 1:**
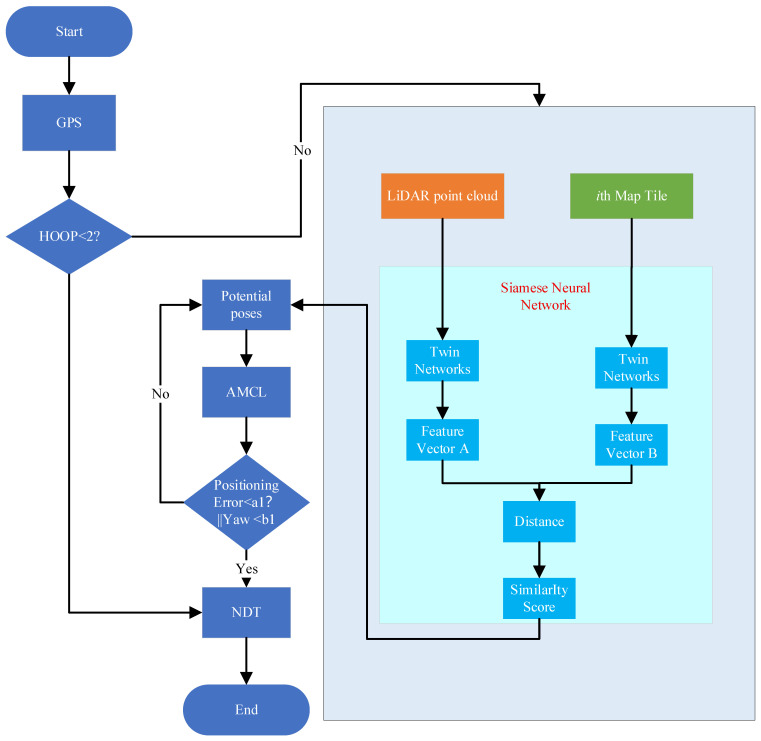
Flowchart of the proposed SNN-based pose initialization and AMCL–NDT refinement framework for GNSS-denied localization.

This study proposes a pose initialization approach based on Siamese network-based feature matching, designed to achieve accurate localization in GNSS-denied environments. The algorithm flowchart is illustrated in Figure 1. When satellite navigation signals are reliable (HDOP < 2), GPS data are used as the initial pose for the NDT algorithm; otherwise, only LiDAR point cloud data are utilized. These point cloud data are fed into the Siamese Neural Network (SNN) to compare with the pre-built map slices, generating a coarse localization candidate set based on the similarity scores. The candidates are then queued and sequentially used as initial pose inputs to the AMCL algorithm for localization refinement, where the AMCL output is compared with a predefined threshold to verify localization success. This process terminates immediately once a valid pose is obtained. The verified pose estimation is used as the initial input for the NDT algorithm for the final refinement, achieving centimeter-level pose optimization.

### 2.1. Self-Built 3D Point Cloud Map

**Figure 2:**
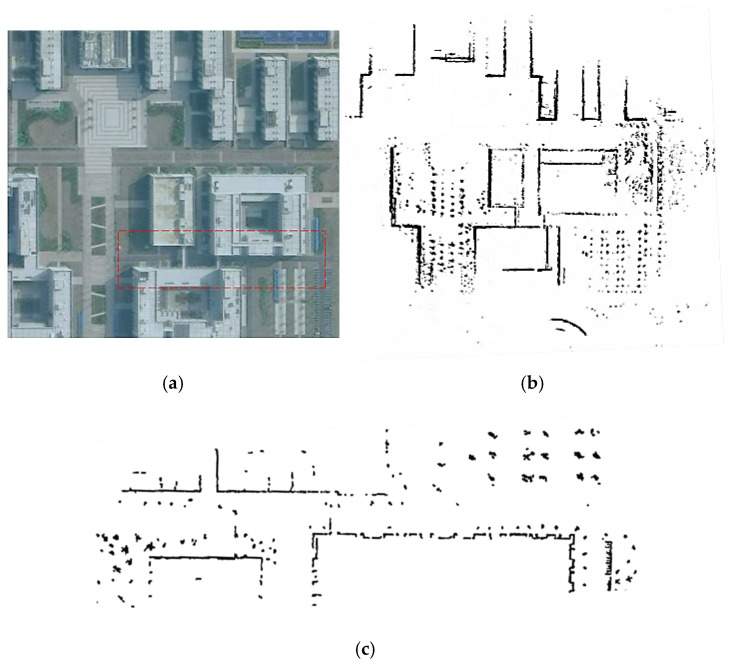
Construction of large- and medium-scale maps: (**a**) satellite image of the data collection area; (**b**) large-scale point cloud map generated using LIO-SAM; (**c**) medium-scale cloud map selected (highlighted by the red box in (**a**)).

A self-built 3D point cloud map was created using LiDAR data collected at the Changzhou Campus of Hohai University (31.6765° N, 119.5713° E). The satellite image is shown in Figure 2a, and the 3D map generated using LIO-SAM is presented in Figure 2b. During preprocessing, ground points were removed using RANSAC-based segmentation, followed by DBSCAN clustering with a radius of 0.2 m and 15 minimum points to eliminate noise, as shown in Figure 2b. These steps reduced registration interference and enhanced matching reliability. The LiDAR-scanned map of the entire area was used as the large-scale scene, while the red-box region in the satellite image served as the medium-scale scene, featuring a denser and more complex point cloud, as illustrated in Figure 2c.

### 2.2. SNN Input Preprocessing

The SNN compares real-time sensor-acquired point clouds with map slices at different candidate locations and produces similarity scores. This section presents the preprocessing steps applied to these two point cloud inputs, ensuring data compatibility while maintaining accuracy and computational efficiency.

#### 2.2.1. Point Cloud Map Preprocessing

**Figure 3:**
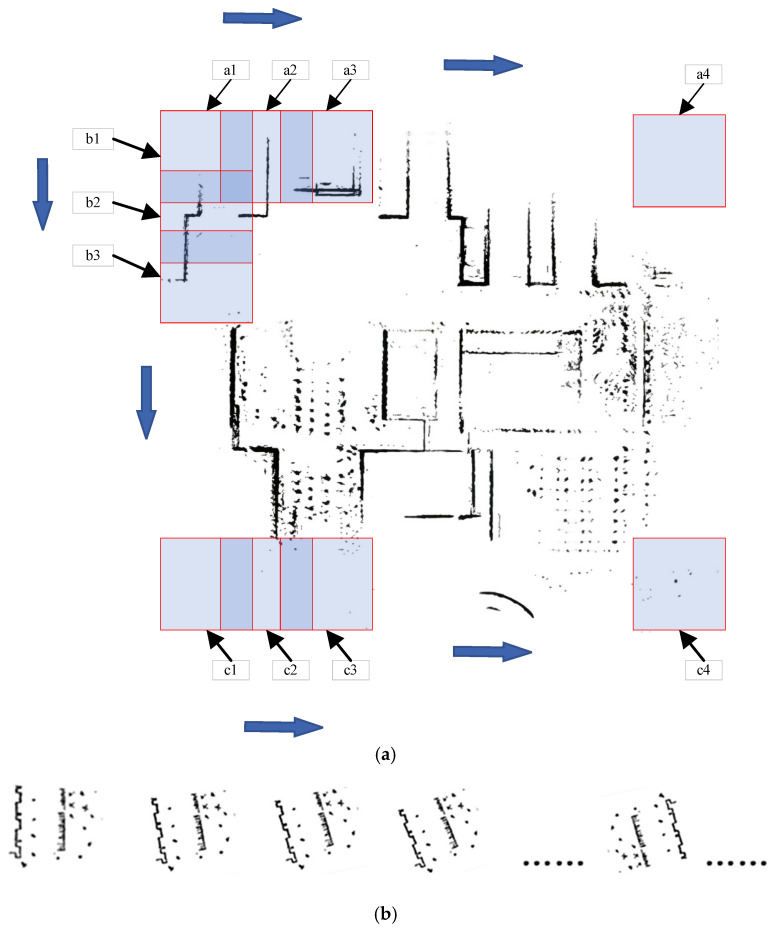
Generation of submap slices with rotations: (**a**) map slices generated via sliding window; (**b**) rotation applied to a single slice for data augmentation.

The purpose of point cloud map preprocessing is to generate local submap slices at different positions with distinct features for matching with real-time LiDAR images. The 3D point cloud data were first projected into a 2D raster map by setting all Z-coordinates to zero using grid resolution of 0.1 m. If the resolution is too large, the point-wise structural information becomes blurred; conversely, if the resolution is too small, inter-point relationships become overly sparse, making feature extraction more difficult. Then, a sliding window was used to generate a series of overlapping slices from the global map, as shown in Figure 3a. A larger window size contains more structural information and is therefore easier to match, but it may reduce localization accuracy; in contrast, a smaller window improves spatial precision but provides fewer discriminative features, increasing matching difficulty. Accordingly, a window size of 15 m, slightly wider than the road width, is selected for the medium-scale map, while a larger window of 30 m is adopted for the large-scale map due to wider road structures. The sliding step was fixed at 2.5 m for both maps. As illustrated in Figure 3b, to improve the rotational invariance of the matching process, each submap was augmented by rotating it at fixed angular intervals to generate a multi-view dataset, facilitating more reliable matching with real-time LiDAR data.

#### 2.2.2. Real-Time Point Cloud Preprocessing

**Figure 4:**
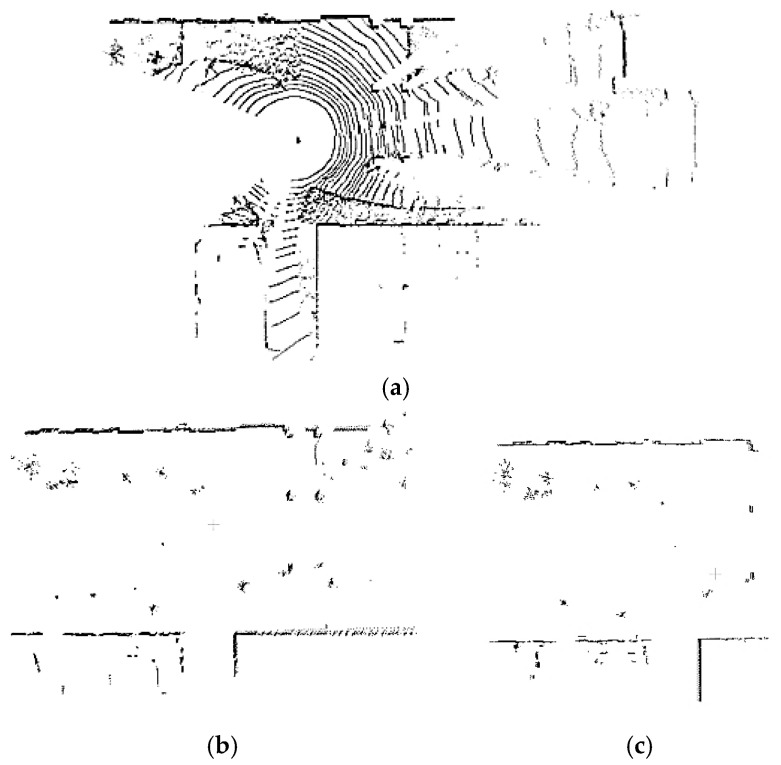
Preprocessing of real-time LiDAR point clouds: (**a**) raw LiDAR point cloud; (**b**) noise and outlier removal using DBSCAN clustering; (**c**) filtering of distant sparse points.

The real-time LiDAR point cloud data (Figure 4a) were preprocessed to ensure compatibility with the map slices. Ground points were first removed using RANSAC-based ground segmentation. Subsequently, DBSCAN clustering was applied to eliminate noise and outlier points (Figure 4b), and distant sparse points were filtered out (Figure 4c). The filtered point cloud was cropped to 15 m × 15 m or 30 m × 30 m to conform to the spatial extent of the submap and projected into a 2D raster map by flattening the Z-coordinates.

### 2.3. Siamese Neural Network-Based Coarse Localization

The proposed coarse localization algorithm is based on a Siamese neural network. Both real-time and global map point cloud data are converted into image representations. Position estimation is performed by comparing sensor-acquired data with pre-built map slices. To ensure the generation of a correct candidate set, the core challenge lies in constructing a Siamese network capable of precisely recognizing image slices of the LiDAR point cloud map.

**Figure 5:**
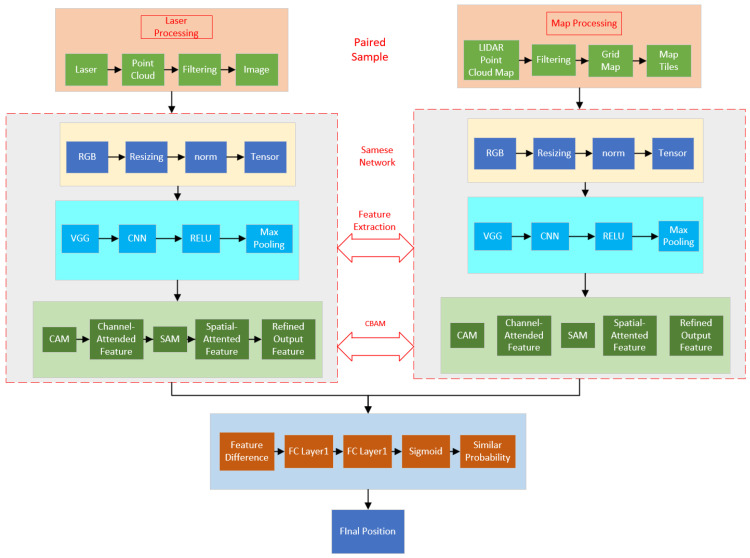
Flowchart of the improved Siamese Neural Network for point cloud slice similarity-based coarse localization.

The architecture of the Siamese network-based image similarity comparison system is shown in Figure 5. It extracts features from two images using weight-sharing neural networks. Feature representation is enhanced through a spatial attention mechanism. The feature differences are then computed and mapped to similarity scores. The underlying principle is that similar images produce similar feature vectors, whereas dissimilar images exhibit larger discrepancies.

Specifically, given a pair of input images, each image is converted into RGB format and resized to 224 × 224. The resized images are processed by the VGG16 feature extraction backbone (13 convolutional and 5 max-pooling layers, with 3 × 3 kernels and ReLU activation function), resulting in a 512 × 7 × 7 feature map for 224 × 224 inputs. To emphasize informative regions such as road features and structural outlines, the feature vectors are refined using the Convolutional Block Attention Module (CBAM), which sequentially emphasizes important channels and highlights spatially relevant structures. This refinement improves the network’s ability to handle variations in rotation and complex scene geometries, resulting in more accurate and robust matching between submaps and real-time LiDAR scans. It is inserted after the feature extraction stage of the Siamese network and before feature difference computation. The channel attention is formulated as:

(1) $$M_{C}(F)=\sigma (MLP(AvgPool(F))+MLP(MaxPool(F)))$$

where *σ*(·) is the sigmoid activation function and *F* represents the input feature map.

The spatial attention is formulated as:

(2) $$M_{S}(F)=\sigma (f^{7\times 7}([AvgPool(F);MaxPool(F)]))$$

where $f^{7\times 7}$ represents a convolution operation with a 7 × 7 kernel.

By incorporating CBAM, the network can automatically focus on information-rich areas and improve discriminative performance. Finally, to quantify image similarity, the network computes the absolute difference *d* between two refined feature vectors $x_{1}$ and $x_{2}$:

(3) $$d=|x_{1}-x_{2}|$$

The absolute difference d is then passed through two fully connected layers: one with 512 neurons (ReLU + Dropout) for nonlinear mapping and dimensionality reduction, and the other with a single neuron producing the similarity score *S*:

(4) S=W2⋅ReLU(W1⋅d+b1)+b2

where $W_{1}$, $W_{2}$, $b_{1}$, and $b_{2}$ are the weights and biases of the two fully connected layers.

The score is converted into a probability score *P* using the Sigmoid function:

(5) $$P=\frac{1}{1+e^{-s}}$$

By sorting the similarity scores *P*, the results with the highest values are retained. The positions corresponding to these high-similarity map slices are selected as coarse localization candidates with coordinates (*x*_i_, *y*_i_):

(6) $$\begin{array}{l} x_{i}=x_{0}+(P_{c}\times R)\\ y_{i}=y_{0}+(P_{r}\times R) \end{array}$$

where (*x*_0_, *y*_0_) is the real-world coordinate of the lower-left corner of the map, *R* is the pixel density in pixel/m of the map, *P*_r_ and *P*_c_ are the row and column indices of the pixel corresponding to the slice center, respectively.

### 2.4. Localization Refinement

The Siamese network-based matching provides an initial position (*x*_1_, *y*_1_) with an error within tens of meters in seconds. This position is refined by AMCL to the meter level and subsequently optimized by NDT to achieve centimeter accuracy.

#### 2.4.1. Adaptive Monte Carlo Localization

AMCL is a particle filter-based algorithm used to estimate the pose (position and orientation) within a known map. It continuously updates and optimizes pose estimation by integrating the motion model with laser scans. The algorithm proceeds as follows:

Initialization: A set of particles {*X*_i_, *W*_i_} is generated according to the initial pose distribution, where *X*_i_ = (*x*_i_, *y*_i_, *θ*_i_) represents the pose of each particle and *W*_i_ denotes its weight.

Prediction: Particle poses are predicted for the next time step using the vehicle motion model. As the vehicle is stationary during pose initialization, the model reduces to preserving the prior state.

Update: Particle weights are updated using the data of LiDAR scans.

Resampling: The particles are resampled based on their weights to retain high-weight samples and discard low-weight ones. Return to the prediction step until the number of retained particles reaches the threshold.

Pose estimation: The final pose is obtained by selecting the highest-weight particle.

Since the vehicle remains stationary and particle weights are updated solely based on sensor data, particle diffusion is absent, and the filter may become overconfident, causing the particle set to collapse into a local optimum. Therefore, covariance or particle dispersion cannot be used to evaluate localization quality. Instead, a likelihood score is employed to measure the matching degree between the LiDAR scan and the map. For each free map cell (*x*, *y*), the distance *d*(*x*, *y*) to the nearest occupied grid is computed, and the likelihood p(*x*, *y*) is defined as:

(7) $$p(x,y)=\frac{1}{\sqrt{2\pi \sigma^{2}}}\times e^{-\frac{d{\left(x,y\right)}^{2}}{2\sigma^{2}}}$$

where standard deviation *σ* controls the decay rate of the likelihood field. The overall likelihood score *L* is the summation of individual cell likelihoods:

(8) $$L=\sum_{m=1}^{k}\log(\frac{1}{\sqrt{2\pi \sigma^{2}}}\times e^{-\frac{d_{k}{\left(x,y\right)}^{2}}{2\sigma^{2}}})$$

The likelihood field model computes the matching probability between LiDAR scan points and map obstacles. A high threshold may cause particle dispersion and poor convergence, while a low threshold may lead to particle degeneration and localization failure. Therefore, a threshold of 2 is selected to balance convergence stability and particle diversity, ensuring sufficient matching between the LiDAR scan and the map in AMCL.

#### 2.4.2. Normal Distributions Transform

NDT partitions the reference point cloud into grids and models each grid as a Gaussian distribution. By optimizing a transformation matrix, it achieves the best matching between the current scan and the reference map. The NDT algorithm proceeds as follows:

1. The reference point cloud is divided into fixed-size grids with Gaussian distributions.
2. The current point cloud $\left\{x_{i}\right\}$ transformed into the reference coordinate frame using a transformation matrix *T*, generating:

(9) $${x_{i}}^{\prime }=T\cdot x_{i}$$

3. The probability score of ${x_{i}}^{\prime }$ is computed as:

(10) $$p({x_{i}}^{\prime })=\frac{1}{\sqrt{{\left(2\pi \right)}^{3}|\Sigma |}}e^{\left(-\frac{1}{2}{\left({x_{i}}^{\prime }-\mu \right)}^{T}\sum^{-1}({x_{i}}^{\prime }-\mu )\right)}$$

where *μ* and Σ denote the mean and covariance matrix of the reference grid, and |Σ| is the determinant of the covariance matrix.

4. Pose optimization is performed using the Newton method to minimize the objective function:

(11) $$score(p)=\sum_{i}e^{\left(-\frac{1}{2}{\left({x_{i}}^{\prime }-\mu \right)}^{T}\sum^{-1}({x_{i}}^{\prime }-\mu )\right)}$$

5. Return to step 2 until the algorithm converges.

## 3. Results

### 3.1. SNN-Based Coarse Localization Experiment

To evaluate the impact of the rotation interval, the Siamese Neural Network in Section 2.3 was trained with augmented datasets generated at various rotation intervals. All experiments were conducted on a PC with an Intel Core i5-10600KF CPU, 32 GB memory, and an NVIDIA GeForce RTX 2060 GPU on Ubuntu 20.04 and CUDA 12.1. The slices were generated as described in Section 2.2.1. The medium-scale map comprises 280 slices, whereas the large-scale map comprises 938 slices. These slices were rotated with intervals between 1.5° and 15° for data augmentation, and the resulting dataset was divided into 80% for training, 10% for validation, and 10% for testing. After training, the probabilities of real-time sensor data being included among the top 5 and top 10 submap candidates ranked by the SNN matching scores were calculated. The results of 100 trials for each interval setting on the medium- and large-scale maps are summarized in Table 1 and Table 2, respectively. For the medium-scale map, smaller rotation intervals result in higher matching success rates due to more complete orientation coverage, but at the cost of larger datasets and longer training time. Compared with the top 5 candidates, the top 10 improve the matching success rate by at least 10%, except for the 1.5° interval. A similar impact of rotation interval is observed for the large-scale map, but it requires 10 candidates to reach a 91% matching success rate, along with extended training time.

**Figure 6:**
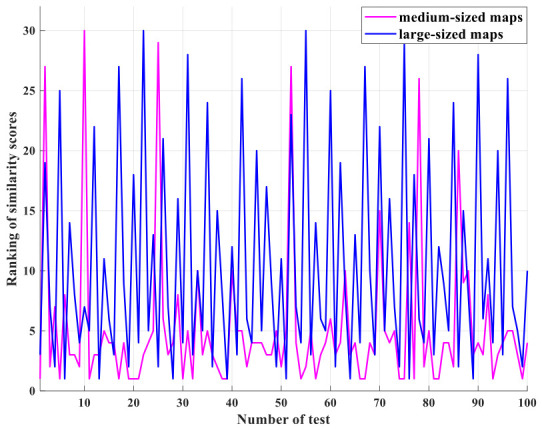
Similarity score rankings of the correctly matched slices at 6° for the medium- and large-sized maps by the SNN.

To achieve a balance between the success rate and the training time, 6° rotation interval is adopted. Similarity score rankings of correctly matched slices at 6° for medium- and large-scale maps using the original SNN is visualized in Figure 6. Results over 100 trials indicate that, for the medium-scale map, the matched ranking fluctuates within the top-5 and top-10 in 81 and 92 cases, respectively. However, the performance degrades significantly on the large-scale map, where only 41 and 62 cases fall within the top-5 and top-10 rankings, respectively, indicating limited robustness of the original SNN under larger-scale environments.

**Figure 7:**
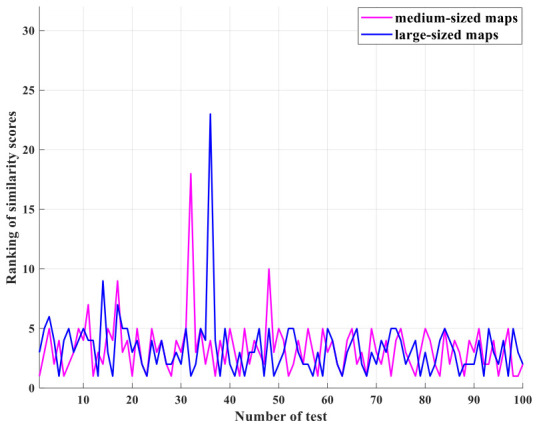
Similarity score rankings of the correctly matched slices at 6° for the medium- and large-sized maps by the CBAM-enhanced SNN.

To further enhance the matching success rate, the attention modules were incorporated into the SNN. The matching success rates of the SNN integrated with different attention mechanisms are summarized in Table 3 and Table 4. Although Squeeze-and-Excitation (SE) and Spatial Transformer Network (STN) achieve success rates of no less than 97% for the top-10 candidates on the medium-scale map, their performance drops considerably on the large-scale map. The self-attention mechanism performs slightly better, showing a smaller decrease on the large-scale map. In contrast, CBAM achieves the best and most stable performance: its success rates of 0.96 for the top 5 candidates and 0.99 for the top 10 candidates remain unchanged across the medium- and large-scale maps. Similarity score rankings of correctly matched slices at 6° for medium- and large-scale maps using SNN enhanced by CBAM is shown in Figure 7. Over 100 trials, correct slices fluctuate within the top-5 and top-10 ranks in 96 and 99 cases for the medium-scale map, respectively, indicating a clear improvement over the original SNN. Similar results are observed on the large-scale map (96 and 99 cases), demonstrating that the improved SNN provides more robust and stable matching performance across different map scales.

**Figure 8:**
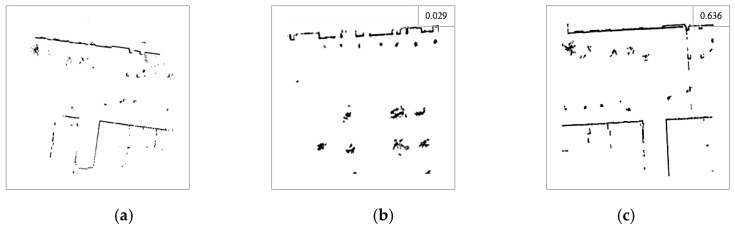

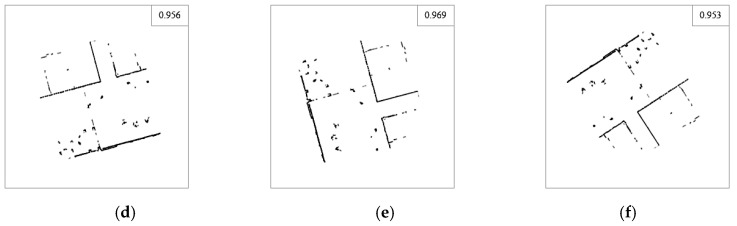
Similarity scores computed by the CBAM-enhanced SNN between the sensor point cloud and map slices from different locations. (**a**) Sensor point cloud; (**b**–**f**) map slices with corresponding similarity scores (shown in the upper-right corner).

Figure 8 presents the similarity scores computed by the CBAM-enhanced SNN between the sensor point cloud in Figure 8a and map slices from different locations (Figure 8b–f). The corresponding similarity scores are shown in the upper-right corner of each image. The low-scoring slices in Figure 8b,c show obvious feature discrepancies, whereas the high-scoring slices in Figure 8d–f corresponding to the correct position with different rotation angles have comparable scores. These results indicate that the SNN assigns high similarity scores to the correct location and is robust to rotational variations.

### 3.2. Localization Refinement Experiment

**Figure 9:**
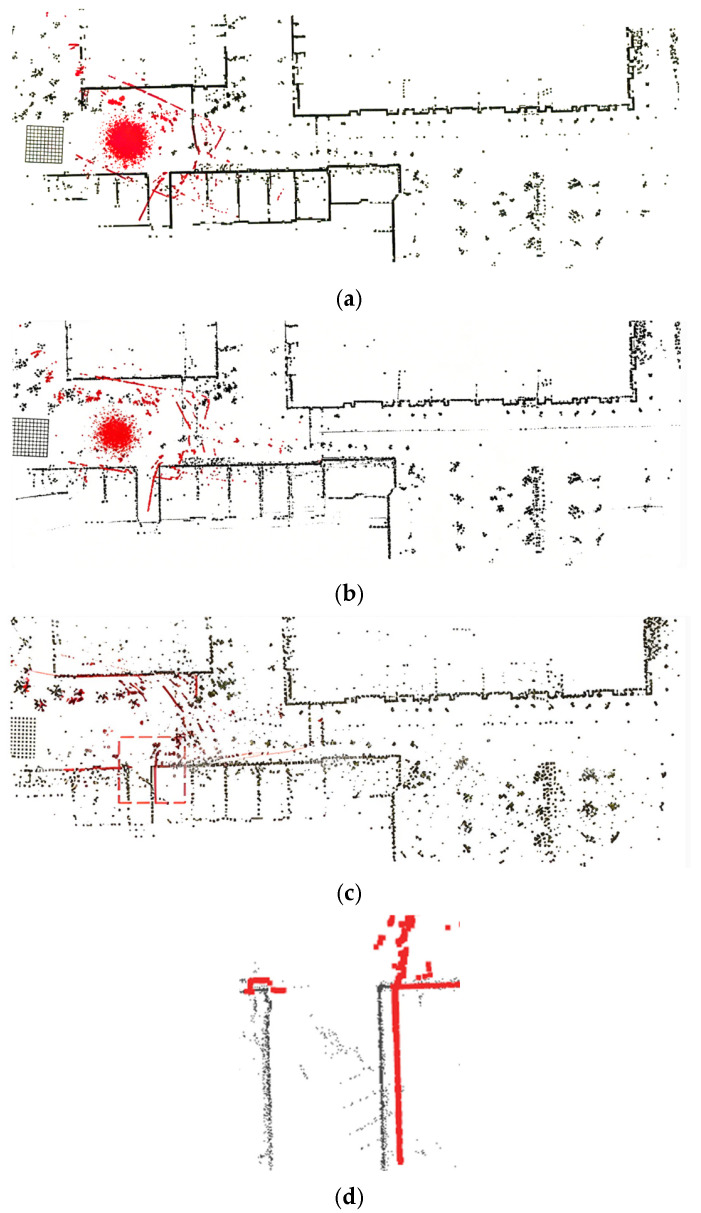
AMCL refinement process. (**a**) Initial coarse position estimated by SNN; (**b**) particle evolution during refinement, (**c**) final localization refinement result after convergence; (**d**) zoomed-in local misalignment.

After obtaining 10 candidate positions from the SNN based on map slice similarity scores, these candidates are used as input for AMCL matching, as described in Section 2.4.1. If the coarse position is correct, AMCL can successfully perform the matching. A typical coarse position, as shown in Figure 9a, is significantly offset from the ground truth, with errors of tens of meters and large orientation deviations. AMCL spreads numerous particles around this coarse estimate. During static localization, the number of particles gradually decreases through iterative refinement, as shown in Figure 9b, until convergence is reached, producing the final position in Figure 9c with meter-level accuracy. At this stage, the sensor point cloud does not perfectly align with the map point cloud, as shown in the zoomed-in view of Figure 9d.

**Figure 10:**
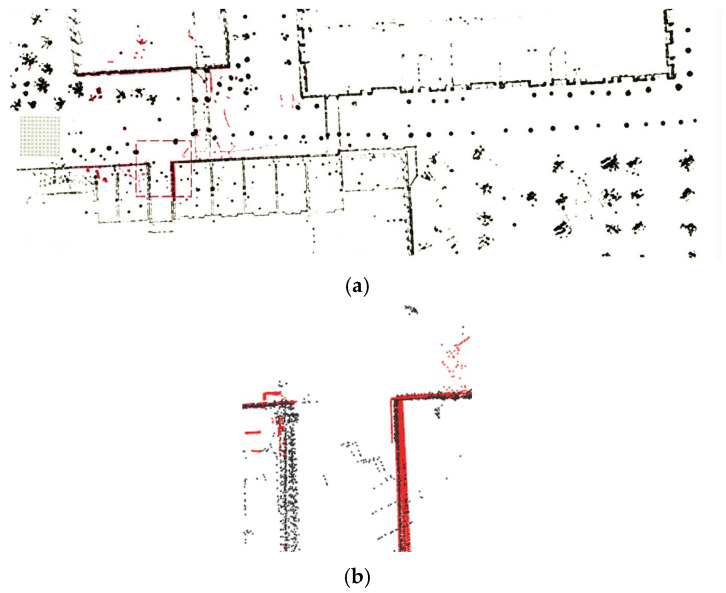
NDT refinement. (**a**) final localization refinement result after convergence; (**b**) zoomed-in local misalignment.

Following the AMCL-refined estimate, NDT computes the statistical properties of grid cells and achieves fast and accurate localization within a few iterations. As shown in Figure 10a, after NDT refinement, as described in Section 2.4.2, the sensor point cloud aligns closely with the map point cloud in both position and orientation. A comparison of the point clouds at the same location in Figure 9d and Figure 10b reveals a substantial improvement in alignment, with the accuracy reaching the centimeter level.

## 4. Discussion

This section evaluates the localization performance of the proposed method through error analysis on both the self-constructed maps and the KITTI dataset.

### 4.1. Localization Error Analysis

**Figure 11:**
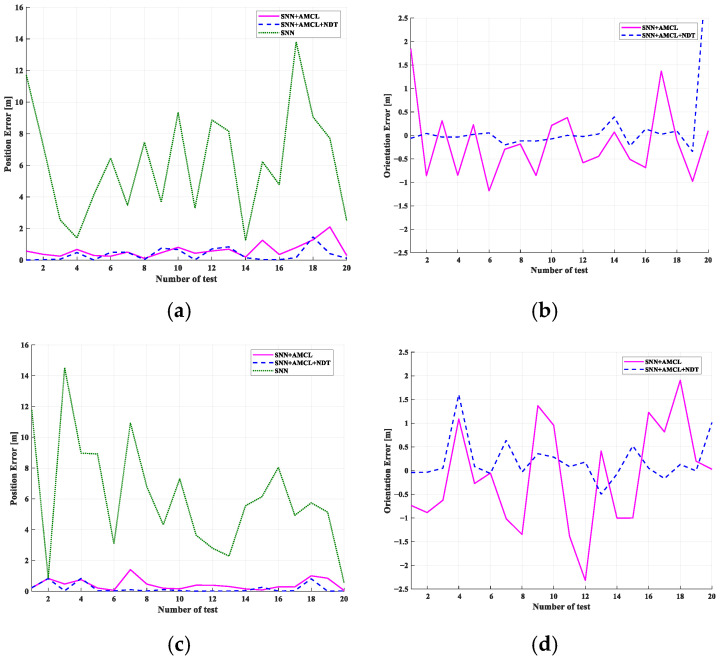
The localization errors of 20 randomly selected positions: (**a**) position errors on the medium-scale map, (**b**) orientation errors on the medium-scale map, (**c**) position errors on the large-scale map, and (**d**) orientation errors on the large-scale map.

The localization errors of the coarse and refined localizations of 20 randomly selected positions in the medium-scale and large-scale maps are shown in Figure 11a,c, respectively. The ground truth was manually obtained by applying rotation and translation to the NDT-based results. For both medium- and large-scale maps, the localization accuracy showed a clear improvement from SNN to AMCL and then to NDT. The SNN coarse localization produced position errors of more than ten meters with significant variations. AMCL effectively reduced these errors by about one order of magnitude, while NDT further reduced the error. In terms of the orientation, as shown in Figure 11b,d, AMCL estimated with errors within 2°, and NDT further refined the angular accuracy. The SNN error curves are absent because it provides only position estimates.

The RMSE values corresponding to Figure 11 are summarized in Table 5. The SNN coarse localization yielded distance RMSEs of approximately 6 m for both map scales. With the subsequent refinements by AMCL and NDT, distance RMSEs were reduced to 0.348 m on the medium-scale map and 0.175 m on the large-scale map. This is primarily due to the randomly selected positions on the large-scale map differing more substantially and containing richer surrounding features. For the orientation, as the elongated road structure in the medium-scale map offers stronger directional cues, the medium-scale map achieved a smaller RMSE after AMCL than the large-scale map, and the final RMSEs reached 0.149° and 0.437°, respectively.

To comprehensively evaluate the performance of the proposed algorithm, comparative experiments were conducted in the medium-scale map against traditional AMCL and Sample Consensus Initial Alignment [39] (SAC-IA), a registration approach based on geometric feature matching. SAC-IA utilizes Fast Point Feature Histograms (FPFH) descriptors with 100 sampling iterations for coarse alignment. Both AMCL and SAC-IA outputs are then used as the initial estimate for NDT-based fine registration. Other mainstream pose initialization methods were not included due to limited applicability for the scenarios. Reliable-loc degrades in feature-deficient environments [40]. OMCL requires careful particle smoothing and may suffer from degeneration in large open spaces [41]. GOToc shows reduced accuracy in sparse scenes [42], while BEVDiffLoc is constrained by limited robustness and generalization ability [43]. Comparison results are summarized in Table 6, where distance and orientation RMSE are calculated from successful localizations. The proposed method achieves a 99% success rate, outperforming AMCL by 27% and SAC-IA by 14%, demonstrating stronger robustness in complex environments. It attains an average distance error of 0.348 m, slightly higher than AMCL but lower than SAC-IA. The orientation error is only 0.149°, reduced by 41.3% compared with AMCL and 95.3% compared with SAC-IA, showing significant improvement in orientation estimation. SAC-IA relies on FPFH descriptors, which are prone to mismatches in repetitive structures and involve high computational cost. In contrast, the proposed method requires only 37 s, reducing runtime by 45.6% compared with AMCL and 72.4% compared with SAC-IA + NDT. These results demonstrate improved efficiency while maintaining high pose initialization performance.

### 4.2. Localization Results in the KITTI Dataset

**Figure 12:**
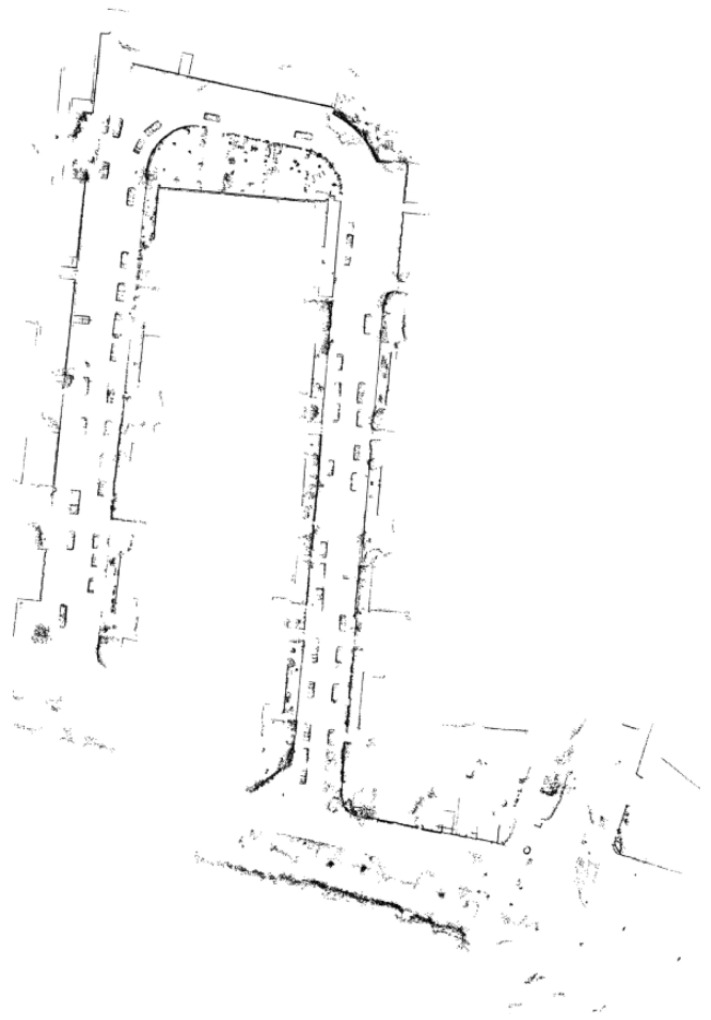
The point cloud map of the selected scene from KITTI dataset.

To evaluate the generalization capability of the proposed algorithm, the KITTI dataset was used for assessment. The point cloud map of the selected scene is shown in Figure 12. This residential environment contains streets, buildings, and a number of parked vehicles. Compared with the campus environment, this scene is more complex and contains many similar regions, posing a substantial challenge for pose initialization. Six locations were randomly selected for testing: (7.626, 20.901, 82.735°), (21.252, 134.262, 59.793°), (74.192, 129.099, −56.967°), (6.752, 4.934, 67.412°), (3.734, 50.150, 47.736°), and (0.093, 91.341, 74.824°). Localization deviations after SNN coarse estimation and subsequent AMCL and NDT refinements are listed in Table 7. The SNN first produced a coarse position with an error below 20 m. AMCL then significantly improved the positional accuracy and provided a relatively rough orientation estimation. After the final NDT refinement, centimeter-level positional accuracy and orientation errors below 0.1° were achieved. These results illustrate that the proposed approach remains effective in more complex and cluttered outdoor environments, demonstrating its applicability across scenes with different structural layouts and object distributions.

## 5. Conclusions

This study presents a high-precision pose initialization framework for GNSS-denied scenarios, leveraging a Siamese Neural Network to provide reliable and efficient coarse position estimates across large-scale maps, which are subsequently refined by AMCL–NDT. The framework demonstrates effective coarse-to-fine localization on both self-collected maps and the KITTI dataset. The proposed framework achieves localization success rates exceeding 99% in two real-world experimental scenarios, with distance RMSEs of 0.175 m and 0.348 m and orientation RMSEs of 0.149° and 0.437°, respectively. However, the current framework still relies heavily on LiDAR sensing and environmental features. Future work will focus on enhancing robustness through multi-sensor fusion (e.g., LiDAR–IMU–vision), generalizing the framework to broader GNSS-denied environments such as tunnels and large-scale indoor warehouse logistics, and improving robustness under degraded LiDAR sensing conditions.

## Figures and Tables

**Table 1:** The success rate using different angle intervals on the medium-scale map.

Angle Interval	Top 5	Top 10	Time (h)
1.5°	0.98	1	168
3°	0.89	0.99	82
6°	0.81	0.92	56
9°	0.77	0.89	41
15°	0.69	0.85	31

**Table 2:** The success rate using different angle intervals on the large-scale map.

Angle Interval	Top 5	Top 10	Time (h)
1.5°	0.76	0.91	78
3°	0.42	0.75	37
6°	0.41	0.62	27
9°	0.06	0.3	19
15°	0	0.48	13

**Table 3:** The success rate of different attention mechanisms on the medium-scale map.

Attention Mechanism	Top 5	Top 10
N/A	0.81	0.92
CBAM	0.96	0.99
SE	0.92	0.97
Self-attention	0.84	0.98
STN	0.66	0.98

**Table 4:** The success rate of different attention mechanisms on the large-scale map.

Attention Mechanism	Top 5	Top 10
N/A	0.41	0.62
CBAM	0.96	0.99
SE	0.4	0.65
Self-attention	0.83	0.96
STN	0.67	0.9

**Table 5:** The localization RMSE of different localization stages.

		SNN	SNN + AMCL	SNN + AMCL + NDT
Medium-scale	*d*	6.153	0.615	0.348
	*θ*	N/A	0.784°	0.149°
Large-scale	*d*	6.112	0.430	0.175
	*θ*	N/A	1.117°	0.437°

**Table 6:** Comparison of localization success rate, RMSE under successful localizations, and runtime among different methods.

	Success Rate	RMSE of *d*	RMSE of *θ*	Running Time
ours	99%	0.348	0.149°	37 s
AMCL + NDT	72%	0.325	0.254°	68 s
SAC-IA + NDT	85%	0.357	3.170°	134 s

**Table 7:** The localization errors of 6 randomly selected positions in KITTI map.

		SNN	SNN + AMCL	SNN + AMCL + NDT
1	*d*	19.849	3.423	0.076
	*θ*	N/A	6.985°	0.035°
2	*d*	10.939	0.376	0.023
	*θ*	N/A	1.259°	0.007°
3	*d*	5.514	1.832	0.018
	*θ*	N/A	10.136°	0.059°
4	*d*	5.139	0.849	0.002
	*θ*	N/A	1.845°	0.061°
5	*d*	8.041	0.290	0.014
	*θ*	N/A	1.372°	0.024°
6	*d*	14.521	0.470	0.029
	*θ*	N/A	1.906°	0.130°

## Data Availability

The raw data supporting the conclusions of this article will be made available by the authors on request.
